# Prediction of emotion distribution of images based on weighted *K*-nearest neighbor-attention mechanism

**DOI:** 10.3389/fncom.2024.1350916

**Published:** 2024-04-17

**Authors:** Kai Cheng

**Affiliations:** School of Artificial Intelligence, Xidian University, Xi'an, China

**Keywords:** image emotions, classification, weighted closest neighbor algorithm, emotional features, abstract paintings

## Abstract

Existing methods for classifying image emotions often overlook the subjective impact emotions evoke in observers, focusing primarily on emotion categories. However, this approach falls short in meeting practical needs as it neglects the nuanced emotional responses captured within an image. This study proposes a novel approach employing the weighted closest neighbor algorithm to predict the discrete distribution of emotion in abstract paintings. Initially, emotional features are extracted from the images and assigned varying *K*-values. Subsequently, an encoder-decoder architecture is utilized to derive sentiment features from abstract paintings, augmented by a pre-trained model to enhance classification model generalization and convergence speed. By incorporating a blank attention mechanism into the decoder and integrating it with the encoder's output sequence, the semantics of abstract painting images are learned, facilitating precise and sensible emotional understanding. Experimental results demonstrate that the classification algorithm, utilizing the attention mechanism, achieves a higher accuracy of 80.7% compared to current methods. This innovative approach successfully addresses the intricate challenge of discerning emotions in abstract paintings, underscoring the significance of considering subjective emotional responses in image classification. The integration of advanced techniques such as weighted closest neighbor algorithm and attention mechanisms holds promise for enhancing the comprehension and classification of emotional content in visual art.

## 1 Introduction

Image data are essentially used for transferring information. The amount of picture data is even increasing at an exponential speed owing to the advent of the Internet (Cetinic and She, 2022; Zou et al., 2023). Because of the fast-paced nature of modern society, people's ability to extract information from photos is also accelerating, necessitating more accuracy and efficiency in identifying image data on the network. Based on this necessity, an effective image processing technique that makes use of computer vision is required for humans to manage and use picture data more effectively.

**Figure 1:**
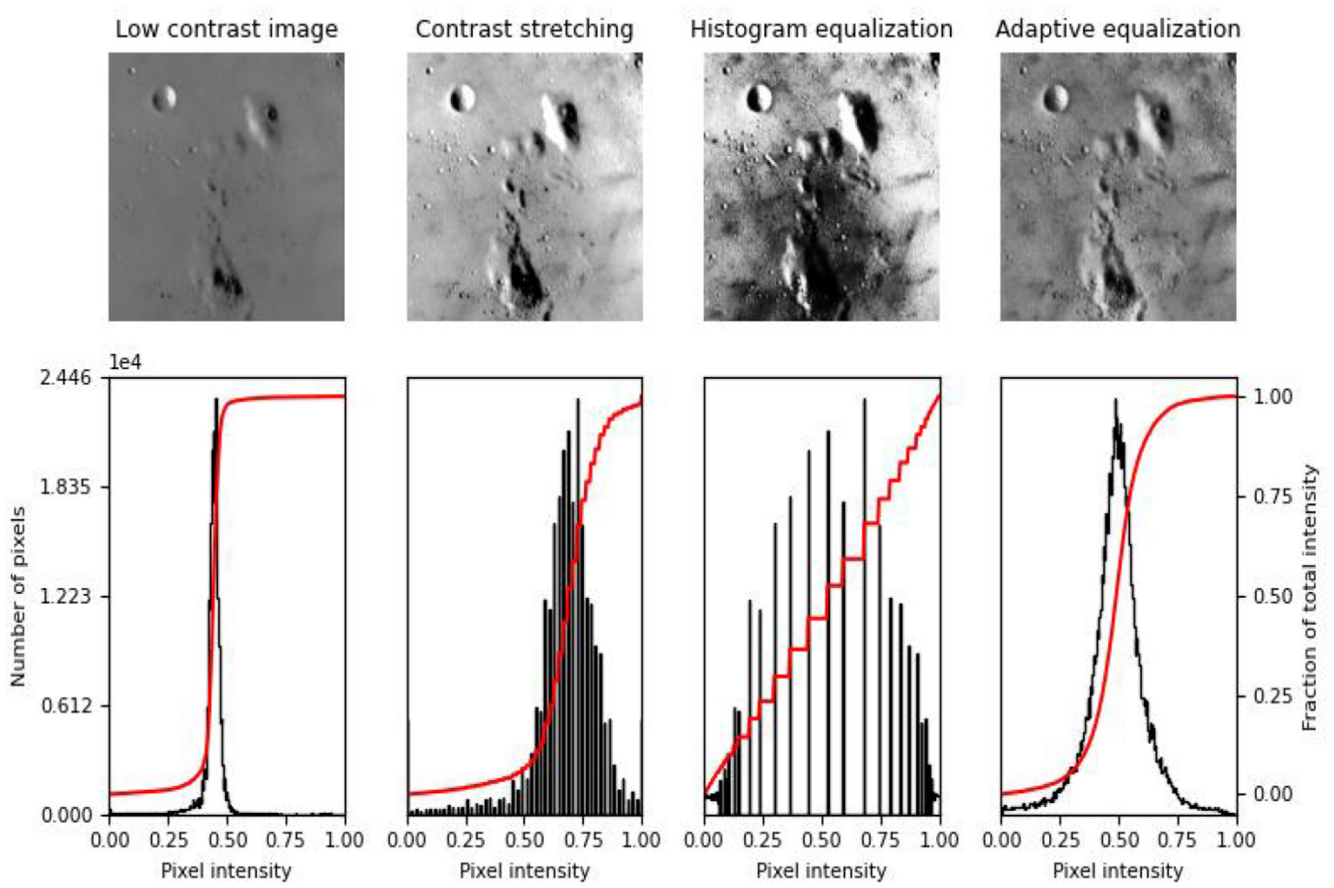
Example of emotional distribution in images.

Sentiment analysis, often called opinion mining, is the process of using natural language processing, text analysis, computational linguistics, and biometrics to systematically unpack subjective information and emotional states. The notion was initially introduced by Yang et al. (2023). Sentiment analysis has gained significant economic and societal significance in the last several years and has been applied extensively in the domains of opinion monitoring (Chen et al., 2023), topic inference (Ngai et al., 2022), and comment analysis and decision-making (Bharadiya, 2023). For monitoring public opinion, the government can make timely policy interventions and accurately determine the direction of public opinion. When it comes to product recommendations, merchants can better understand user needs and suggestions by gauging user satisfaction with product evaluations and enhancing product quality. In the finance domain, trending financial topics can even be used to predict stock direction. Furthermore, sentiment analysis is frequently used for various tasks involving natural language processing. To increase the accuracy of the system, more exact terms for sentiment expression are chosen for machine translation (Chan et al., 2023) by evaluating the sentiment tendency of the input text. The pixel density extraction of the image information is shown in Figure 1.

Various classification techniques will be broken down into different levels for the sentiment analysis task: output results will categorize the methods into sentiment intensity classification and sentiment polarity classification; granularity of the processed text will divide them into three research levels: word level, sentence level, and chapter level; research methodology will separate them into unsupervised learning, semi-supervised learning, and supervised learning, and so on. The majority of the conventional sentiment classification algorithms employ manually created feature selection techniques for feature extraction, such as the maximum entropy model (Chandrasekaran et al., 2022), plain Bayes (Wang et al., 2022), support vector machines (Zhao et al., 2021a), and so on. However, these techniques have limitations, such as being labor-intensive, time-consuming, and hard to train. As a result, they are not well-suited for use in the current large-scale application scenarios.

With advancements in machine learning, research efforts (Milani and Fraternali, 2021) led to the development of deep learning methods that give neural networks a hierarchical structure. This development subsequently resulted in an explosion of deep learning research. Feature learning, at the heart of deep learning, uses hierarchical networks to convert unprocessed input into more abstract and higher-level feature information. With its superior learning capacity to optimize automated feature extraction, deep learning has produced remarkable research achievements in recent years in the domains of speech recognition, picture processing, and natural language processing. The application of deep learning techniques to text sentiment analysis has gained popularity as a natural language processing study area. Among these techniques, Song et al. (2021) used a convolutional neural network to classify text emotion for the first time, and the results were superior to those of conventional machine learning techniques.

The study of human eyesight is where attention mechanism first emerged. According to cognitive science, humans have a tendency to ignore other observable information in favor of focusing on a certain portion of the information based on the demand imposed by the information processing bottleneck. The primary objective of attention mechanism is to efficiently separate valuable information from a vast quantity of data. To understand the word dependencies inside the phrase and grasp the internal structure of the sentence, the self-attention mechanism—a unique form of attention mechanism—is incorporated into the sentiment classification job. To establish an accurate and efficient technique for sentiment analysis based on deep learning technology and self-attention mechanism, this study examines the present technical issues in the field of sentiment analysis from the standpoint of the real demands of sentiment analysis.

## 2 Related studies

Natural language processing has attracted extensive research attention (McCormack and Lomas, 2021) because it introduced the idea of sentiment analysis. There are three prominent methods for conducting sentiment analysis at present: the sentiment dictionary approach, the classical machine learning approach, and the deep learning approach.

Experts must annotate the sentiment polarity of the text's terms in order for researchers to perform sentiment analysis based on sentiment dictionary. Based on semantic rules and sentiment dictionary, researchers compute the text's sentiment score and determine the sentiment tendency. Among these researchers, Toisoul et al. (2021) demonstrated positive findings on a multi-domain dataset by expanding the domain-specific vocabulary by extracting subject terms from the corpus using latent Dirichlet allocation (LDA) modeling based on the pre-existing sentiment lexicon. Peng et al. (2022) used the point mutual information (PMI) technique to assess the similarity of adjectives in WordNet. The polar semantics (ISA) approach was then used to generate numerous fixed sentence constructions in order to examine the target text sentiment tendency. To create a Chinese microblogging sentiment dictionary, Liu et al. (2021a) first identified microblogging sentences using information entropy and then filtered network sentiment terms using the sentiment-oriented pointwise mutual information (SO-PMI) method.

Ding et al. (2021) introduced the idea of the primary word and used weight priority calculations to determine the text's semantic inclination degree. These developments paved the way for accomplishing more difficult sentiment analysis tasks. The approach based on sentiment dictionary has the benefit of being more accurate in classifying text at the word or phrase level. However, the system migration is not good, and the sentiment dictionaries are often geared to certain domains. These days, one of the most popular techniques for sentiment analysis is classical machine learning-based techniques. Using simple bag-of-words features from a movie review dataset, Yang et al. (2021) was the first to use machine learning techniques to the sentiment binary classification issue and produced superior experimental outcomes. Utilizing Twitter comments as test data, Roy et al. (2023) classified emotions into six categories—happiness, sadness, disgust, fear, surprise, and anger—and employed plain Bayes for text sentiment analysis. The data were processed with consideration for lexical and expression features, leading to a high classification accuracy. To address the sentiment classification problem, Sahoo et al. (2021) merged a genetic algorithm with simple Bayes, and the results of the experiments indicated that the combined model outperformed the individual models. To extract rich sentiment data and include them in the basic feature model, Liu et al. (2021b) used machine learning techniques with numerous rules, which increased the classification result in microblog sentiment classification trials. In order to complete the study of sentiment analysis, Sampath et al. (2021) included semantic rules into the support vector machine model. The experiment confirmed that the support vector machine model with the inclusion of semantic rules performed better in the sentiment classification task. Deeper text semantic information is hard to learn, even while machine learning-based techniques enhance the sentiment classification performance and lower the reliance on sentiment lexicon.

Text sentiment analysis based on deep learning has garnered a much interest from academics at both national and international levels due to its superior performance in the fields of picture processing and natural language processing. Zhang et al. (2023) used deep neural network training to create the Collobert and Weston (C&W) model, which was then used to perform well on natural language processing tasks including sentiment classification and lexical annotation. To demonstrate the efficacy of single-layer convolutional neural networks (CNNs) in sentiment classification tasks, Zhao et al. (2021b) combined different sizes of convolutional kernels with maximum pooling and performed comparison tests on seven datasets. The study employed convolutional neural networks for sentiment analysis tasks. A number of recurrent neural networks, including recurrent neural network (RNN), multiplicative RNN (MRNN), recursive neural tensor network (RNTN), and others, were progressively suggested by Szubielska et al. (2021). The RNTN model, for example, uses a syntactic analysis tree to determine word sentiment and then outputs the sentence's sentiment classification result in the form of word sentiment summation. To tackle the sentiment analysis problem utilizing a long short-term memory (LSTM) network with an expanded gate structure, which increases the model's flexibility, Li et al. (2022) employed Twitter comments as the experimental data. RNNs were utilized by Zhou et al. (2023) to model texts by taking into account their temporal information. Li et al. (2023) achieved outstanding results in a sentiment classification test by modeling utterances using a tree LSTM model to approximate the sentence structure. By segmenting a text according to sentences, obtaining vectors through convolutional pooling operation, and then inputting them into LSTM according to temporal relations to construct a CNN-LSTM model and apply it to the task of sentiment analysis, Alirezazadeh et al. (2023) primarily addressed the issue of temporal and long-range dependencies in a chapter-level text. Teodoro et al. (2023) constructed an experimental minimal convolutional neural network (EMCNN) model using microblog comments as the experimental data, combining lexical and emoji characteristics. The model produced experimental findings that outperformed the benchmark model's performance.

## 3 Attention given

**Figure 2:**
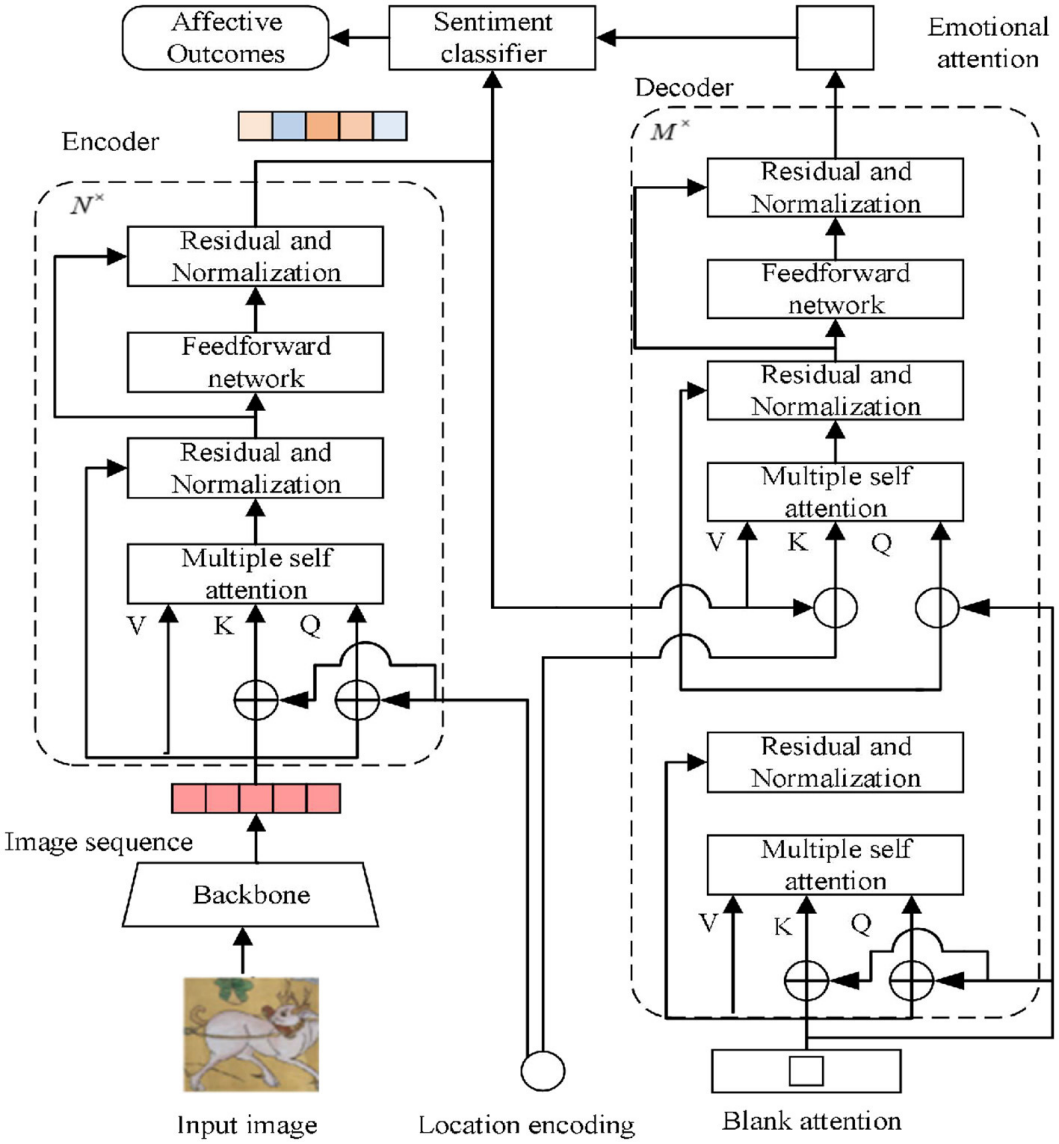
Model of this article.

We propose an emotion classification method based on the attention mechanism that sets blank attention in the decoder and fuses the output sequence of the encoder to learn the image semantics to guide the model to learn the image emotion more accurately and reasonably via the learning mechanism of the decoder. This method is intended to address the characteristics of small numbers of abstract painting samples and rich image semantics. Figure 2 depicts the general flowchart of the procedure used in this article, along with the encoder–decoder architecture, the emotion classification module, and the backbone network for extracting picture feature sequences.

### 3.1 Image sequence generation

Since the encoder anticipates a sequence as input, the abstract painting dataset in this study has been uniformly normalized, meaning that its length and width are 224 and its number of channels is 3. To extract the image's features, the image is supplied into the backbone network. The residual network has a strong feature learning ability and adapts to the characteristics of the backbone convolutional network architecture. In this study, ResNet-50 is adopted as the backbone network to solve the network degradation problem brought by fewer samples of abstract paintings to simplify the model training parameters of this article to a certain extent, improve the training efficiency, and carry out comparative experiments with the residual network variant in the ablation experiments, and to assess the influence of the backbone network on the model accuracy rate (Ahmad et al., 2023). The abstract painting dataset is generated by the backbone network to generate canonical image features with a length and width of 7 and a channel count of 256 and is spread into a one-dimensional sequence, resulting in an image sequence of length 49 and a channel count of 256 to be fed to the encoder.

### 3.2 Encoders

By adjusting the number of encoder layers, the model demonstrates the significance of global image-level self-attention, guarantees that there is no appreciable loss of accuracy when *N* = 6, and prevents an increase in training difficulty brought on by the addition of too many parameters. This article adopts the position coding method of detection transformer (DETR), which uses the sine and cosine functions to encode the positions of rows and columns of the parity channel of the abstract painting feature map, adapting to the sequence input of the encoder–decoder architecture (Ahmad and Wu, 2023). The encoder–decoder architecture is not sensitive to the order of the image sequence and does not have the ability to learn the sequence position information. The calculation for the position coding as shown in Equation (1):


(1) $$f{\left(x\right)}^{i}=\left\{ \begin{array}{l} \sin\left(\frac{x}{10000^{i/128}}\right),i=2k\left(k\in [0,127]\right)\\ \cos\left(\frac{x}{10000^{i/28}}\right),i=2k+1\left(k\in [0,127]\right) \end{array}\right.$$

where *x* is the row and column spread value of point (*p, q*) and *i* is the channel of the feature map. For a feature map with a length and width of 7 and a channel count of 256, respectively, the row and column position encoding on the point with a channel of 10 and a coordinate value of (1,2) is sin [((1 × 7) +2)/(1,000,010/128)] and sin [((2 × 7) +1)/(1,000,010/128)], respectively, and the position encoding of the remaining image sequences of the channels is computed by this rule. Encoding finally generates a one-dimensional feature sequence with a length of 49 and a channel count of 256 with position information.

The *Q, K, V* in the encoder is a one-dimensional sequence of a fixed length of 49 and a channel count of 256, which is used as sentiment weights in translating the image sequence and ordering its position in each encoding session. As the model learns the feature dependencies between image sequences, the multi-head self-attention module supports the model by reinforcing the original features with sequence global information. This support enables the model to learn discriminative features for sentiment classification. The original image sequence serves as the input for the first coding layer, and the input for each succeeding layer is the image sequence encoded in the preceding layer. The picture feature sequences are given to the decoder after being encoded and learned by many coding layers of the encoder, avoiding the issue of delayed network convergence and poorer accuracy brought on by the increased depth of the model.

### 3.3 Decoders

The blank attention in this study has the same format as the feature sequence of the model input, that is, a sequence with a fixed length of 49 and a channel count of 256. Similar to the encoding phase, the blank attention is weighted as a query statement with *Q, K, V* of the first self-attention layer in the decoder, but at this point, the blank attention does not need to focus on the location information. At each decoding stage, the multi-head attention module transforms the blank attention sequences and generates the output of the attention sequences with weights by avoiding the problem of slower model convergence through the residuals and normalization module.

The attention sequence with weights from the upper layer and the output sequence from the encoder are fed into the second self-attention layer. This study uses the same sine and cosine functions in the decoder as in the encoder to encode the position of the weighted attention sequences from the upper layers since the output sequence of the encoder contains positional information and needs to accommodate its positional connection. The positional encoding of the picture sequence for each channel is calculated for the weighted attention sequence of length 49 and a channel count of 256. This positional encoding is applied to the rows and columns of the parity channels. Ultimately, a weighted attention sequence of length 49 and a channel count of 256 with position information are obtained. It is combined with the output sequence of the encoder as a query statement and weighted with *Q, K, V* from the second layer in the decoder. In each decoding stage, the output sequence of the encoder is translated, and the sequence positions are sorted.

### 3.4 Classification of emotions

**Figure 3:**
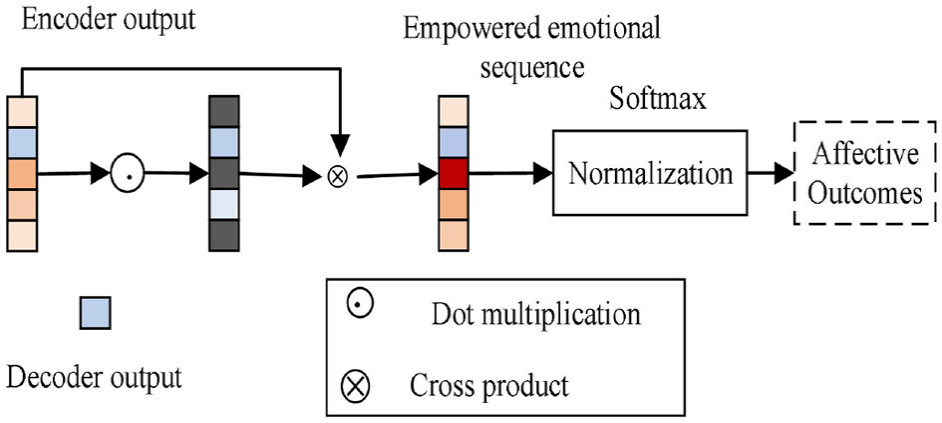
Emotional classification.

Figure 3 depicts the emotion classification module. The sentiment classification module combines the output sequences of the encoder and decoder to produce weighted sentiment sequences, which suppress redundant sentiment information in the model, direct the model to concentrate on deep and shallow sentiment information, and improve the model's ability to classify sentiment. The fully connected layer is used to map the weighted sentiment sequences, and the cross-entropy loss is minimized to produce stable sentiment classification results (Ahmad et al., 2021). The normalized exponential function is used to calculate the probability value of each type of sentiment; the abstract painting sentiment predicted by the model has the highest probability value.

The normalized exponential function is as shown in Equation (2):


(2) $$\begin{array}{l} S_{i}=\frac{e^{i}}{\overset{12}{\underset{j=1}{\sum }}e^{j}} \end{array}$$

where *S*_*i*_ is the normalized value of a particular sentiment and computation max(*S*_*i*_) is the abstract painting sentiment label predicted by the model.

One popular loss function for handling classification difficulties is the cross-entropy function, which is primarily used to quantify the difference between two probability distributions. The cross-entropy loss function is as shown in Equation (3):


(3) $$\begin{array}{l} Loss=\frac{1}{batch\text{\_}size}\underset{i}{\sum }\overset{12}{\underset{c=1}{\sum }}y_{ic}\log_{2}\left(p_{ic}\right) \end{array}$$

Each term in the cross-entropy function is *p* and *q* and *p* indicates the true probability distribution and *q* represents the predicted probability distribution. The cross-entropy function describes the difference between the two probability distributions. For the special case, the cross-entropy function of the binary classification problem, there are a total of two terms, i.e., probability distributions of classes 0 and 1, and there is *p*(0) = 1−*p*(1), so we can get the expression for the binary classification cross entropy loss function, where *y*_*ic*_ is the true value and *p*_*ic*_ is the probability of the predicted value.

## 4 Image preprocessing

### 4.1 Datasets

The abstract dataset, which includes 280 abstract paintings, was created by Machajdik. These paintings are better suited for challenges requiring the prediction of emotion distribution because they simply feature colors and textures and not any clearly discernible objects. The 230 participants in the dataset expressed their emotions by identifying these 280 photographs, with an average of 14 people doing so. The final sentiment category is determined by which of these sentiment markers received the most votes. Due to the ambiguity of emotions, several categories may have extremely similar or identical numbers of votes, making the classification process unclear. Therefore, the ratio of votes for each emotion category is used as a probability distribution to form a probability distribution of emotions corresponding to the image, as shown in Figure 3.

### 4.2 Feature extraction

Since abstract paintings contain only colors and textures and do not generate emotions through specific objects, the features extracted are emotional features based on the theory of artistry.

#### 4.2.1 Color histogram

Artists use colors to express or trigger different emotions in observers, and extracting color histograms from color features is a common and effective method. The color histogram space *H* is defined as Equation (4):


(4) $$\begin{array}{l} H=\left[h\left(0\right),h\left(1\right),...h\left(L_{k}\right)\right],\overset{K}{\underset{k=1}{\sum }}h\left(L_{k}\right)=1 \end{array}$$

where *h*(*L*_*k*_) denotes the frequency of the *k*th color. The similarity of the color histograms of the two images are measured using the Euclidean distance as shown in Equation (5):


(5) $$\begin{array}{l} D\left(H_{s},H_{d}\right)=\left[{\left(H_{s}-H_{d}\right)}^{T}{\left(H_{s}-H_{d}\right)}^{1/2}\right] \end{array}$$

#### 4.2.2 Itten comparison

Itten successfully used the strategy of color combination by defining seven contrast attributes. Machajdik used seven contrast attributes such as light and dark contrast, saturation contrast, extension contrast, complementary contrast, hue contrast, warm and cool contrast, and simultaneous contrast of images as the emotional characteristics of artistry theory.

As in the case of light and dark contrast, the image is segmented into *R*_1_, *R*_2_...*R*_*N*_, small chunks using the watershed segmentation algorithm, and the average *h*_*n*_ (Chroma) *b*_*n*_ (Brightness) *s*_*n*_ (Saturation) is calculated for each chunk. Calculation *b*_*n*_ belongs to five fuzzy luminance: $\left\{\begin{array}{l} \mathrm{Very}\mathrm{Dark}(VD)\text{,}\mathrm{Dark}(D)\text{, middle }(M),\\ \mathrm{Light}(L)\text{,}\mathrm{Very}\mathrm{Light}(VL) \end{array}\right\}$ affiliation function as shown in Equations (6–10).


(6) $$\text{VD}=\left\{ \begin{array}{l} \;1b_{n}\;⩽\;21\\ \frac{39-b_{n}}{18}\;21\;<\;b_{n}\;⩽\;39\\ 0 \end{array}\right.$$

(7) $$\text{D}=\left\{ \begin{array}{l} \frac{b_{n}-21}{18}\;21<b_{n}\leq 39\\ \frac{55-b_{n}}{16}\;39<b_{n}\leq 55\\ \;0 \end{array}\right.$$

(8) $$\text{M}=\left\{ \begin{array}{l} \frac{55-b_{n}}{16}\;39<b_{n}\leq 55\\ \frac{b_{n}-55}{13}\;55<b_{n}\leq 68\\ \;0 \end{array}\right.$$

(9) $$L=\{\begin{array}{c} \frac{b_{n}-55}{13}\;55<b_{n}\leq 68\\ \frac{84-b_{n}}{16}\;68<b_{n}\leq 84\\ 0 \end{array}$$

(10) $$\text{VL}=\{\begin{array}{c} \frac{84-b_{n}}{16}\;68<b_{n}\leq 84\\ 1\;b_{n}>84\\ 0 \end{array}$$

Thus, a 1^*^5 dimensional vector for each small block of image *R*_1_, *R*_2_...*R*_*N*_ is obtained, and for the whole image, the light/dark contrast is defined as Equation (11):


(11) $$\text{B}(\text{i})={\left[\frac{1}{\sum_{n=1}^{N}R_{n}}\sum_{n=1}^{N}R_{n}{\left(B_{n}(i)-\bar{B}(i)\right)}^{2}\right]}^{1/2}$$

where *i* = 1, …, 5, *R*_*n*_ is the number of pixels in the split block.

In this way, the vector expression of the contrasting attributes of the images is obtained as features, and the similarity of the different images is calculated by the Euclidean distance.

The Itten model is also used to determine whether or not an image is harmonic, and it can also be used to identify an image's emotional expression. Select three to four of the image's prominent colors, connect them to the colors on the Itten hue wheel, and if they form a positive polygon, the image is harmonic. To determine the dominant chromaticity of an image, make a histogram of its N colors. Ignore the colors with a proportion of < 5%. The harmony of a polygon can be assessed by comparing its internal angles to those of a square polygon built from the same number of vertices.

#### 4.2.3 Texture

The main idea behind the statistical approach to texture analysis is to symbolize textures by the randomness of the distribution of gray levels in a graph. We define *z* as a random variable representing the gray levels, *L* as the maximum gray level of the image, *Z*_*i*_ as the number of pixels with gray level *i*, 01 denotes the gray level histogram, and with respect to *z*, the nth order moments are calculated as shown in Equation (12):


(12) $$\begin{array}{l} u_{n}\left(z\right)=\overset{L}{\underset{i=0}{\sum }}{\left(Z_{i}-m\right)}^{n}p\left(z_{i}\right) \end{array}$$

$\begin{array}{l} m=\overset{L}{\underset{i=0}{\sum }}z_{i}p\left(z_{i}\right) \end{array}$ is the mean value of z.

The second-order moments are more important in texture description; it is a measure of grayscale contrast, where $R=\frac{1}{1+u_{2}\left(z\right)}$ indicates the smoothness of the image, and a smaller value of *u*_*n*_(*z*) corresponds to a smaller *R* value, indicating that the smaller the value of R, the smoother the image.

### 4.3 Weighted *K*-nearest neighbor sentiment distribution prediction algorithm

Assuming that there are *M* sentiment categories *C*_1_, ⋯ , *C*_*M*_ and *N* training images, *x*_1_⋯ , *x*_*N*_ (which also denote the corresponding features of the images) use $\text{p}={\left\{P_{n1},\cdots ,P_{nm}\cdots ,P_{nM}\right\}}^{T}$ to denote the sentiment distribution of *X*_*n*_, where *P*_*nm*_ denotes the probability that *x*_*n*_ expresses a sentiment of *c*_*m*_, and for each image, there is $\overset{M}{\underset{m=1}{\sum }}P_{nm}=1$. Assuming that *y* is a test image, the goal of this study is to find the sentiment distribution $\text{p}={\left\{P_{1},\cdots ,P_{M}\right\}}^{T}$ of y, i.e., as shown in Equation (13).


(13) $$\begin{array}{l} \text{f}({\left\{x_{n},p_{n}\right\}}_{n=1}^{N},y)\to \text{p} \end{array}$$

Training sets that are very far away have little effect on *y*. Considering that including all training sets can slow down the run and irrelevant training samples can also mislead the algorithm's classification, the effect of isolated noise samples can be eliminated by taking a weighted average of the *K*-nearest neighbors.

Weighted *K*-nearest neighbor option denotes only the drizzle functions corresponding to the *K* training images that assign the larger weights to the closer nearest neighbors. denotes the sentiment distribution of the *K* training images nearest to the test image, which is considered as a basis function, and the sentiment distribution *P* of the test image *y* is computed by performing a distance-weighted summation of the basis function, i.e.,


(14) $$\begin{array}{l} P=\frac{\overset{K}{\underset{k=1}{\sum }}s_{k}p_{k}}{\overset{K}{\underset{k=1}{\sum }}s_{k}} \end{array}$$

where *s* is the similarity between the test sample and the training sample, as shown in Equation (15).


(15) $$\begin{array}{l} s=e\left(-\frac{d\left(x_{k},y\right)}{\beta }\right) \end{array}$$

where *d* is the Euclidean distance and β is the average distance of *y* from the training images.

Algorithm: Weighted *K*-nearest neighbor sentiment distribution prediction algorithm.

Input: Training set (*x*_*n*_, *p*_*n*_), test set *y*.

Output: Sentiment distribution *p* for the test set.

1. Calculate the distance *d* between the test set image *y* and each image in the training set.
2. Select the first *k* images *x*_1_...*x*_*k*_ that are closest to *y* in the increasing order of distance.
3. $\beta =\frac{1}{k}\sqrt{{\left(x_{1}-y\right)}^{2}+\cdots +{\left(x_{k}-y\right)}^{2}}$ is brought into Equation (14) in order to compute the similarity *s*.
4. Calculate the sentiment distribution of the test image *y* $P=\frac{\overset{K}{\underset{k=1}{\sum }}s_{k}p_{k}}{\overset{K}{\underset{k=1}{\sum }}s_{k}}$.

## 5 Experimentation and analysis

### 5.1 Landscape image

**Figure 4:**
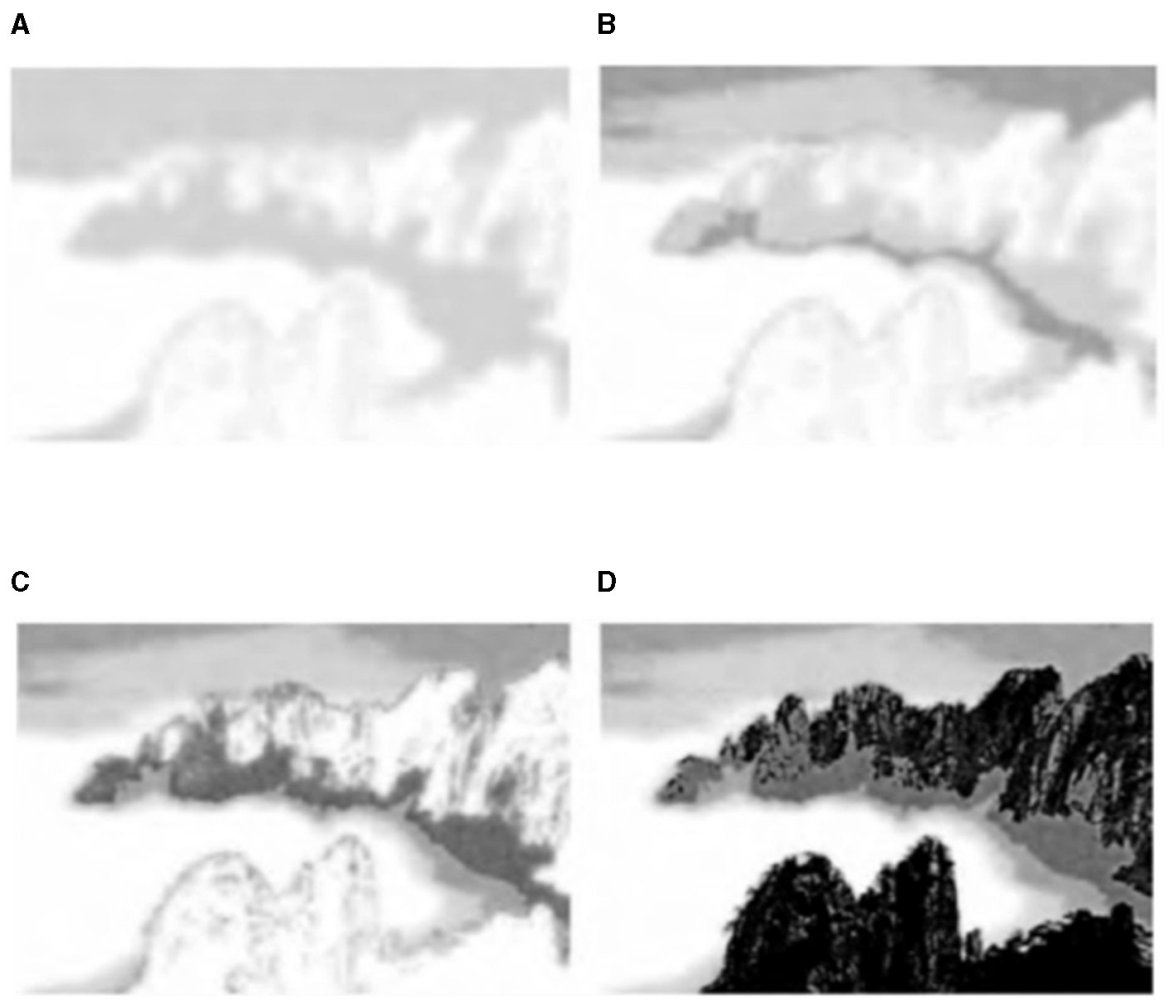
Layer stacking process effect and ink simulation results. **(A)** One grayscale layer overlay effect. **(B)** Two grayscale layer overlay effect. **(C)** Three grayscale layer overlay effect. **(D)** Seven grayscale layer overlay effect.

Experiments on a large number of landscape images (resolution of ~1 million pixels, downloaded from “Baidu images”) to achieve the simulation of ink and wash painting and to achieve a more satisfactory simulation effect. Figure 4 represents the algorithm from shallow to deep ink “drawing” simulation process: Figure 4A shows the layer effect, Figures 4B, C represent the first two layers and the first three layers of the superposition effect, and Figure 4D shows the seven-layer superposition effect, that is, the final eight-ink effect [*p*_*u*_(*u*_*j*_) = (0.2, 0.15, 0.15, 0.15, 0.15, 0.15, 0.15, 0.15, 0.15, 0.15, 0.15, 0.15, 0.15, 0.15, 0.15, 0.15, 0.15, 0.15, 0.15, 0.15, 0.15 and Figure 4A for the 00 layer effect. 15, 0.15, 0.15, 0.11, 0.06, 0.03)].

**Figure 5:**
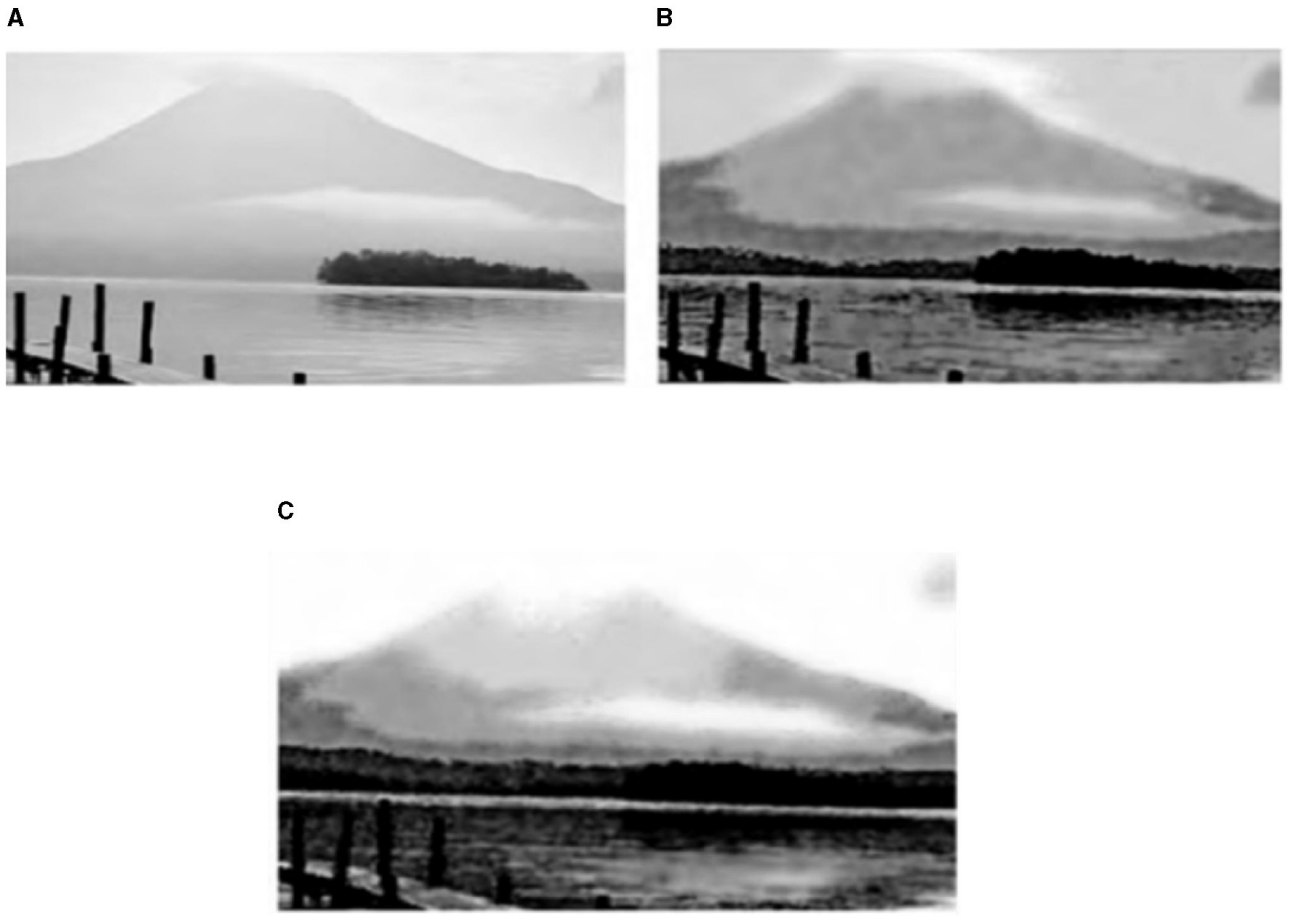
Comparison of direct eighth order color reduction and histogram prescribed ink simulation 1. **(A)** Landscape original 2. **(B)** Direct eighth order color reduction link effect. **(C)** Histogram normalization link effect.

**Figure 6:**
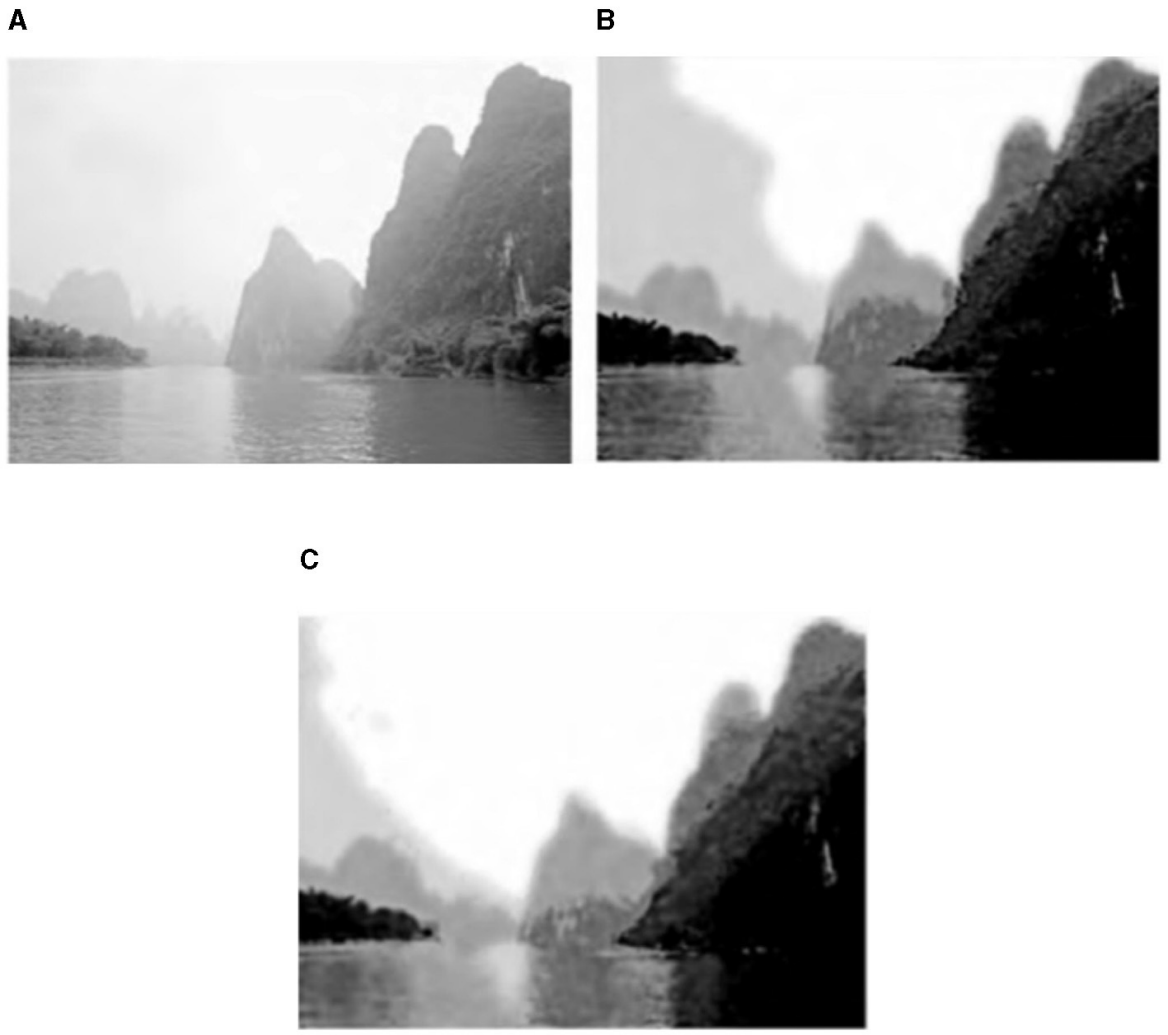
Comparison of direct eighth order color reduction and histogram prescribed ink simulation 2. **(A)** Landscape original 3. **(B)** Direct eighth order color reduction link effect. **(C)** Histogram normalization link effect.

In the algorithm, the histogram specification serves to preset the weight of each ink color and enhance the recognition of the inked area. Figures 5, 6 show a comparison of the ink simulation experiments for two other sets of landscape images and reveal the role of histogram specification in the simulation effect. Figures 5B, 6B show the effect of the algorithm based on direct eighth order grayscale color reduction, and Figures 5C, 6C show the effect of the algorithm based on histogram specification. The values of *p*_*u*_(*u*_*j*_) were [0.2, 0.15, 0.15, 0.15, 0.15, 0.15, 0.11, 0.06, 0.03] and [0.3, 0.125, 0.15, 0.125, 0.125, 0.1, 0.05, 0.025]. The direct eight-order color reduction approach is governed by the color values of the original diagram, which is easily the source of the imbalance of the weight of each ink color and the lack of distinctiveness, as can be seen from the comparison of the two sets of diagrams. The histogram specification method can better control the amount of ink colors and especially strengthen the weight of *Gray*(0) (i.e., white area). Ink simulation has a better sense of hierarchy and differentiation. In summary, the algorithm in this article simulates the ink effect of the landscape map through the method of layer simulation ink overlay, the simulation map has a strong sense of hierarchy, and the layers of ink can be integrated with each other and also has a natural paper-ink penetration effect.

### 5.2 Abstract paintings

The existing sentiment classification networks ResNet and Swin Transformer and their variants are compared under the sentiment classification accuracy metrics in order to assess the effectiveness of the model in this article. The encoder–decoder structure with various numbers of layers is set up for this article's method; the one-layer encoder–decoder structure is defined as Tiny and the six-layer encoder-decoder structure is defined as Base. By training five batches of experimental findings and averaging them as the final results of the experimental data, five rounds of cross-validation were used to test the models. To accelerate the convergence of abstract painting sentiment classification, each model is fine-tuned based on the ImageNet pre-trained model, using the Adam W optimizer with a weight decay of 0.1/30 epoch and an initial learning rate of 0.0001, and trained based on the NVIDIA RTX 2080Ti.

The actual Naxi Dongba abstract paintings were gathered from the literature on Na xi abstract paintings, and the abstract paintings were divided into four categories based on the subject matter of the painting's creation. For instance, in the abstract painting data set shown in Table 1, the figures, ghosts and monsters, animals, and plants are shown from left to right, and the abstract paintings were divided into 12 different emotion categories based on the emotions they conveyed.

**Table 1:** Example of part of the abstract painting dataset.

**Emotion**	**Painting theme**			
	**Character**	**Ghosts**	**Animal**	**Plant**
Negative	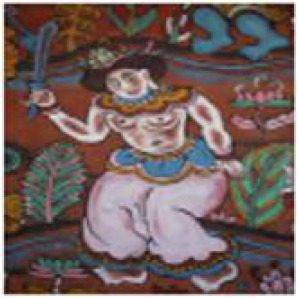	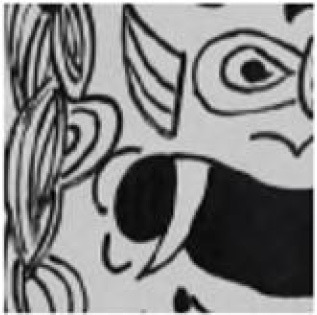	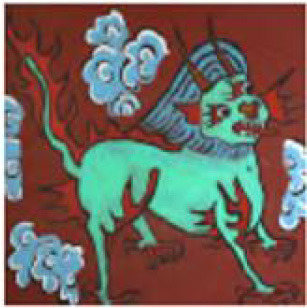	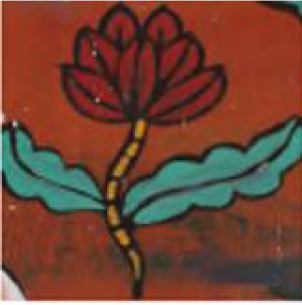
	Be cautious	Aversion and resistance	Anxiety and tension	Faint and wilting
Neutral	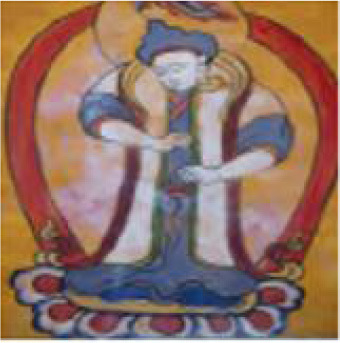	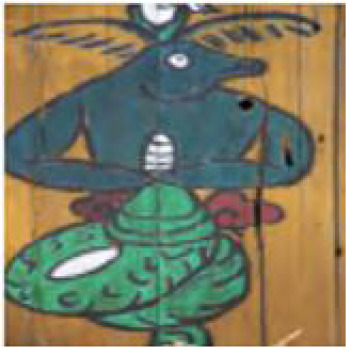	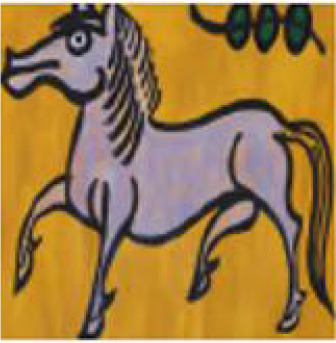	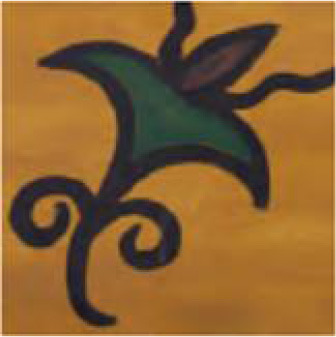
	Harmony and friendliness	Neutral and Pure	Be pragmatic and responsive	Impartial and impartial
Positive	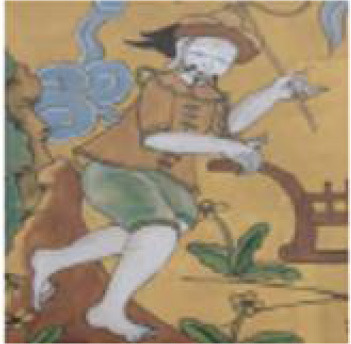	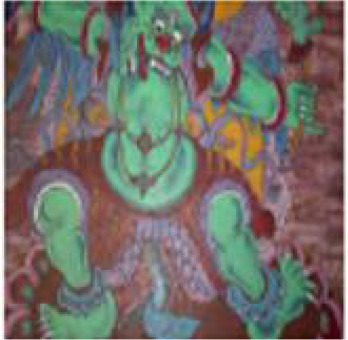	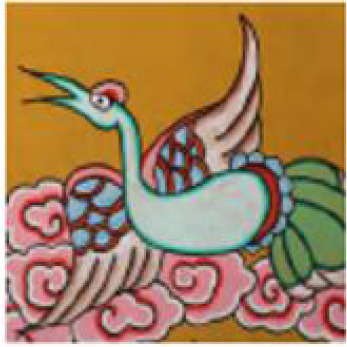	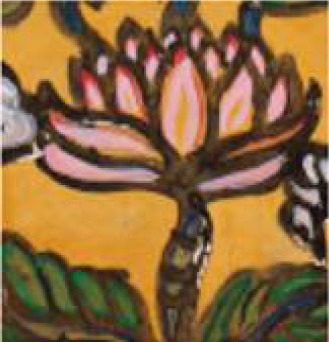
	Diligent and simple	Enthusiastic and proactive	Elegant and gentle	Beautiful and graceful

ResNet50 was used as the backbone network in order to extract image features and tested on the test set for sentiment classification of abstract paintings.

The experimental findings in Table 2 demonstrate that the algorithm presented in this article is superior to ResNet, Swin, and their variation network topologies for the job of sentiment recognition for abstract paintings. Established sentiment classification techniques like ResNet-101 and Swin-B achieved classification accuracies of 71.4 and 73.2%, respectively, whereas this article's method-Tiny and method-Base produced the best classification outcomes with classification accuracies of 74.3 and 80.8%, respectively.

**Table 2:** Abstract painting emotion classification experiment.

**Model**	**Classification accuracy (%)**
ResNet-18	64.6
ResNet-34	68.4
ResNet-50	70.3
ResNet-101	71.4
Swin-T	70.1
Swin-S	72.7
Swin-B	73.2
Vit-T	72.7
Vit-B	76.8
Method of this article—Tiny	74.3
Method of this article—Base	80.8

The existing sentiment classification techniques do not account for the deeper sentiment elements that are buried in abstract paintings; instead, they focus on predicting the sentiment labels of abstract paintings while neglecting their linguistically complex and emotionally varied properties. The method in this article, in contrast, uses blank attention in the decoder and fuses the encoder's output sequence while learning the semantics of the abstract painting image as the emotion attention through the decoder's decoding learning mechanism. As a result, the method employed in this study is able to achieve a higher classification accuracy rate.

This study first conducts ablation experiments on the backbone network, compares a variety of Res Net variants to replace the backbone network, and keeps the structure of this article's model unchanged for the experiments in order to assess the impact of the number of parameters of the backbone network on the accuracy of sentiment classification. The results of the experiments are shown in Table 3.

**Table 3:** Backbone network experiment.

**Method**	**Backbone**	**Parameter quantity (M)**	**Accuracy (%)**
1	ResNet-18	29	72.5
2	ResNet-34	39	76.7
3	ResNet-50	42	80.8
4	ResNet-101	61	81.9

The model parameter amount was 42 M and the classification accuracy was 80.8% when ResNet-50 was used as the backbone network. The number of model parameters was cut to 29 M with the use of ResNet-18, however the model's classification accuracy dropped by 8.3%. ResNet-34, on the other hand, reduced the number of model parameters by 3 M while increasing the classification accuracy of the model by 4.1% when utilized as the backbone network. The number of model parameters rises by 19 M when ResNet-101 is used as the backbone network, yet the classification accuracy increases by 1.1%. In this article, choosing ResNet-50 as the backbone network ensures that there is no significant decrease in the accuracy rate and avoids the increase in training difficulty due to the introduction of too many parameters.

**Figure 7:**
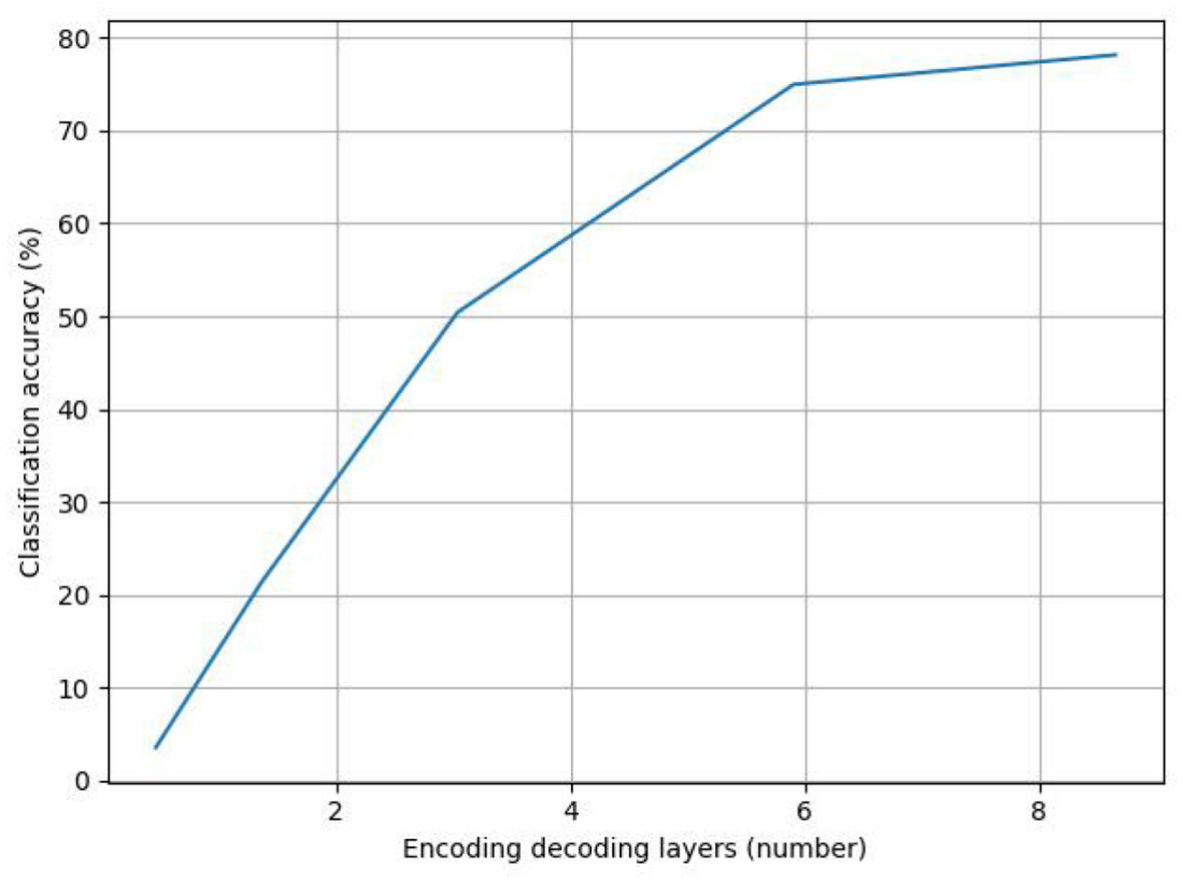
Analysis of the number of encoding and decoding layers.

Figure 7 displays the line graph of the experimental analysis of the number of coding–decoding layers; as the number of coding–decoding layers increases, the model's accuracy gradually increases, suggesting that adding more coding–decoding layers can, to a certain extent, increase the accuracy of the classification of the emotions in abstract paintings. The model uses six coding–decoding layers to achieve 80.8% classification accuracy, avoiding the overfitting issue that results from the stacking of coding–decoding layers. However, as the number of coding–decoding layers increases, the improvement in accuracy eventually slows down and becomes flat.

To prove the effectiveness of this article's attention mechanism for classifying the emotions of abstract paintings, two types of ablation models are set up to eliminate the decoder and encoder outputs, based on keeping the backbone network of the model as ResNet-50: The attention mechanism setup is not used in the ablation model, which eliminates the output of the decoder. Instead, the model uses the coded sequence output from the encoder as the basis for emotion classification. The classifier then normalizes the coded sequence to determine the likelihood of outputting emotion labels through the full connectivity layer spreading. The fully linked layer disperses the coded sequence, and the normalization of the classifier determines the likelihood of producing emotion labels; The attention mechanism established in this research is kept in the ablation model that removes the encoder output, and the model continues to mine the picture semantics of the abstract paintings without fusing the encoder output with the attention mechanism. In Table 4, the experimental findings are displayed.

**Table 4:** Ablation experiment.

**Method**	**Decoder output**	**Encoder output**	**Accuracy (%)**
1	X	√	74.3
2	√	X	77.9
3	√	√	80.8

When the encoder is used to help classify the abstraction of drawing sentiments, the accuracy of sentiment classification decreased after eliminating the decoder output by 6.5%, showing higher classification accuracy than that of the ResNet-50 classification model; however, after eliminating the encoder output, the accuracy of sentiment classification of the ablation model decreased by 2.9%, which is higher than that of the ResNet-101 classification model and close to that of the ResNet-50 classification model. This finding shows that the attention mechanism in this study can help the model recognize abstract paintings' emotions more accurately by acting as a facilitator.

In this study, we used a full convolutional network to calculate the emotional weights of the model, visualize the weight heat map of the model, and simultaneously highlight and locate the regions in the heat map that significantly influence the expression of emotion.

**Figure 8:**
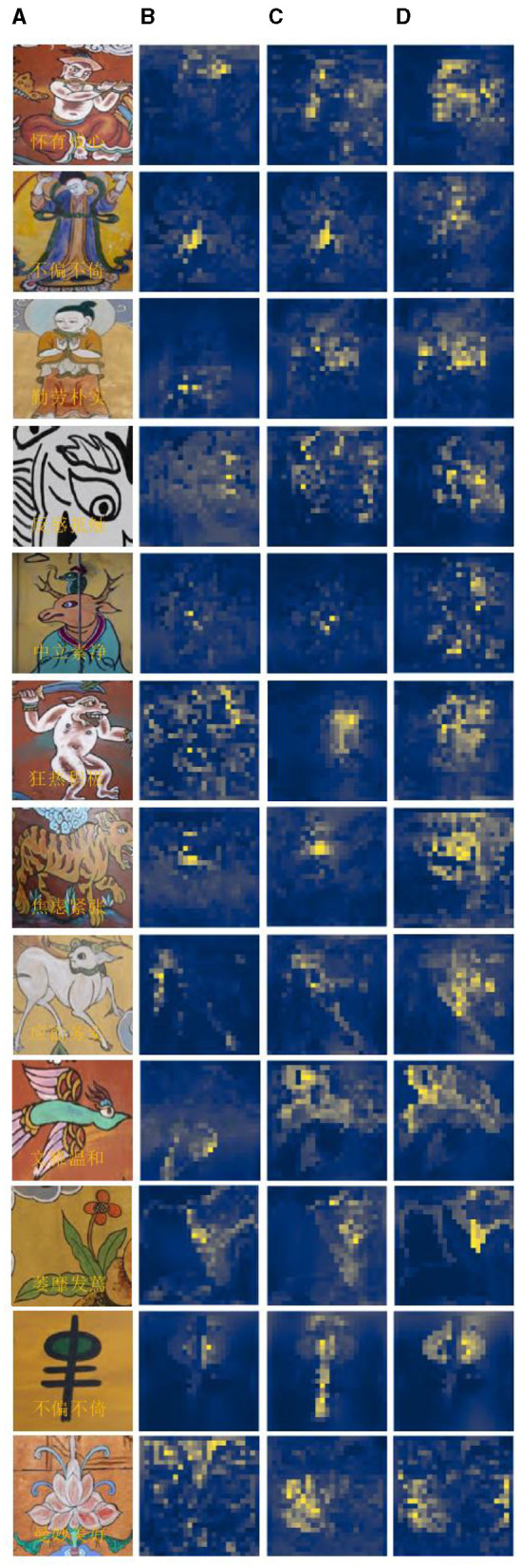
Presentation of visualization results: **(A)** initial image; **(B)** elimination decoder; **(C)** elimination encoder; and **(D)** model of this article.

Figure 8A provides an illustration of an abstract painting's original image, which is tagged with the predicted emotions derived from the image by the model test and contains 12 emotions as determined by the experimental data, respectively. The ablation model produced by the elimination decoder is depicted in Figure 8B, with loose regions of attention and unfocused regions of interest in the model's heat map; the regions of interest for abstract paintings of various subjects also differ significantly from one another. Figure 8C demonstrates that, despite being more compact, the model heat map's zone of interest suffers from ambiguous regions of interest and incorrect localization. It is also unresponsive to a smaller percentage of the neutral emotion image. The focus in the figure paintings is on the behavior and movements of the Dong ba figures, and the areas highlighted by the model labeled colors in the different image emotions correspond to the areas of the abstract paintings where the figures are holding arms, dancing, and making gestures, respectively. Figure 8D shows the model heat map of this article, which has a more concentrated region of interest and more stable localization. For emotionally complex animal paintings, the model expands the emotional expression to the animal's body area; for the plant paintings, the color highlighting points out the plant petal area, which corresponds to the plant's budding or blossoming gesture. In the ghost paintings, the model heat map focuses on the ghost behavior and action area.

The visualization experiments demonstrate the comparison experiments of the ablation model and the region of interest of the model described in this article. They also show how the relationship between the abstract painting emotion attention and the image emotion learned by this article model is more intimate and how this has a more immediate effect on the results of the emotion classification. It demonstrates how well the model in this study extracts the emotion from images of abstract paintings, making it more appropriate for classifying the emotions of abstract paintings.

### 5.3 Predictive distribution

**Figure 9:**
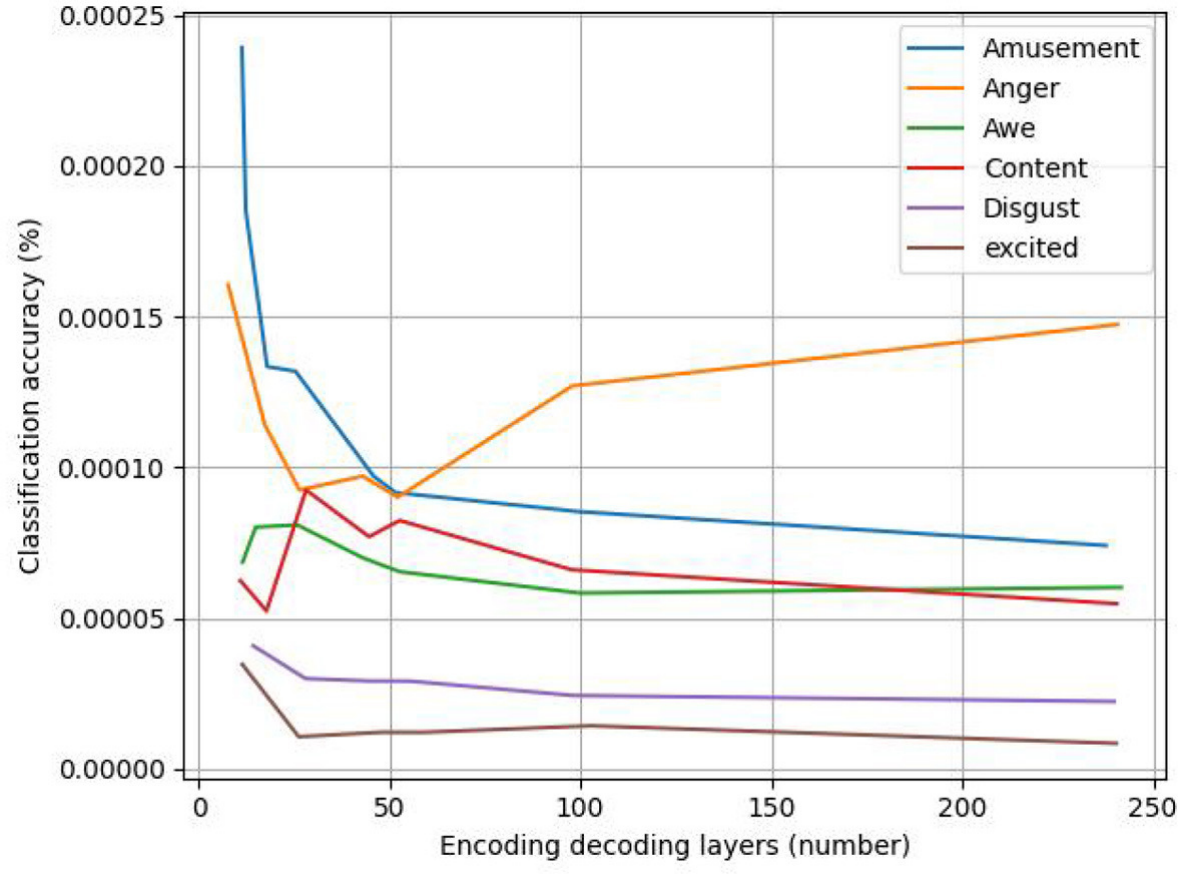
Effect of different *K*-values on the prediction of sentiment distribution.

The effect of different values of *K* (*K* = 5, 10, 20, 40, 50, 100, 252 using 10-fold cross-validation, where *K* = 252 is the global weighting of the training set) on the prediction of the sentiment distribution in a weighted *KKN* is shown in Figure 9.

In this example, the optimal *K*-value is affected by the sentiment category, and considering the average performance, it is considered that the best prediction is achieved at *K* = 40, 50, which outperforms the global weighting, and when *K* = 252, all the training images are used for distribution prediction.

## 6 Conclusion

The majority of early algorithms employed for sentiment classification were based on shallow machine learning and extract features using manually constructed feature selection techniques that have weak generalization ability, require extensive training times, and entail high labor costs. Because of its superior learning capacity to optimize feature extraction and prevent the flaws of manual feature selection, deep learning has produced positive research outcomes in the field of text sentiment categorization. The attention mechanism's primary objective is to swiftly separate valuable information from a vast amount of data. When applied to the sentiment classification task, it is capable of identifying word dependencies within sentences and identifying the internal organization of the sentence. Using a weighted closest neighbor technique, we provide a novel approach in this study to predict the discrete sentiment distribution of each picture in an abstract painting. Testing shows that the attention mechanism-based classification algorithm achieves a better classification accuracy of 80.7% when compared to state-of-the-art techniques, thereby resolving the issues of rich material and difficulties in identifying the emotions shown in abstract paintings. Nevertheless, there are several drawbacks to the attention mechanism in this article, such as its incapacity to create the positional link between objects and scenes in abstract paintings. Furthermore, it is restricted by the dataset on abstract paintings and is unable to sufficiently address the issues of imprecise sentiment categorization and imprecise attention learnt from datasets that are made publicly available. Future research methods might thus expand the sentiment dataset to a broader picture data domain and further expand the abstract painting sentiment classification system to a multimodal level in order to overcome these problems.

## Data availability statement

The original contributions presented in the study are included in the article/supplementary material, further inquiries can be directed to the corresponding author.

## Author contributions

KC: Writing – original draft.
